# Natural Convection in a Differentially Heated Square Enclosure with a Solid Polygon

**DOI:** 10.1155/2014/617492

**Published:** 2014-06-02

**Authors:** R. Roslan, H. Saleh, I. Hashim

**Affiliations:** ^1^Faculty of Science, Technology & Human Development, Universiti Tun Hussein Onn Malaysia, 86400 Parit Raja, Batu Pahat, Johor, Malaysia; ^2^School of Mathematical Sciences, Faculty of Science & Technology, Universiti Kebangsaan Malaysia (UKM), 43600 Bangi Selangor, Malaysia; ^3^Solar Energy Research Institute, Universiti Kebangsaan Malaysia (UKM), 43600 Bangi Selangor, Malaysia; ^4^Department of Mathematics, Faculty of Science, King Abdulaziz University, P.O. Box 80257, Jeddah 21589, Saudi Arabia

## Abstract

The aim of the present numerical study is to analyze the conjugate natural convection heat transfer in a differentially heated square enclosure containing a conductive polygon object. The left wall is heated and the right wall is cooled, while the horizontal walls are kept adiabatic. The COMSOL Multiphysics software is applied to solve the dimensionless governing equations. The governing parameters considered are the polygon type, 3 ≤ *N* ≤ *∞*, the horizontal position, 0.25 ≤ *X*_0_ ≤ 0.75, the polygon size, 0 ≤ *A* ≤ *π*/16, the thermal conductivity ratio, 0.1 ≤ *K*_*r*_ ≤ 10.0, and the Rayleigh number, 10^3^ ≤ Ra ≤ 10^6^. The critical size of the solid polygon was found exists at low conductivities. The heat transfer rate increases with the increase of the size of the solid polygon, until it reaches its maximum value. Here, the size of the solid polygon is reaches its critical value. Further, beyond this critical size of the solid polygon, will decrease the heat transfer rate.

## 1. Introduction

Natural convection heat transfer in differentially heated enclosures from side or below has received considerable attention over the past few decades, largely due to a wide variety of applications, which include solar collector technology, energy storage, nuclear reactor and technology. Another practical application of natural convection is encountered when an obstacle such as an inserted object is placed inside the enclosure. In applications involving building energy components, such as walls, windows, or an electronic enclosure, the inserted solid object may reduce the flow, thereby reducing the heat transfer rate across the enclosure. On the other hand, heat transfer may be enhanced if the solid object has a relatively high thermal conductivity.

House et al. [1] firstly examined the effect of a centered heat-conducting object on natural convection heat transfer in a vertical enclosure for various Rayleigh number, Prandtl number, object size, and ratio of thermal conductivities. They obtained that the heat transfer may be enhanced or reduced by a square object with a thermal conductivity ratio less or greater than unity. Later, Liu and Phan-Thien [2] included a radiation effect. Oh et al. [3], Deng and Tang [4], and Zhao et al. [5] visualized the problem by the heatlines concept. Merrikh and Mohamad [6] found that placing the solid objects near to the walls reduces the rate of heat transfer due to the blockage effects, but placing low conductor objects far from the boundary layer region may enhance the rate of heat transfer compared with enclosures without obstacles. Ha et al. [7] used a spectral multidomain methodology to handle a square object located at the center and concluded that the Rayleigh number for the fluid and temperatures fields to become nonsymmetrical and time dependent depends on the thermal boundary conditions of the the object. Bhave et al. [8] reported an existence of the critical size of the adiabatic object. The heat transfer increases with the increase of the size of the object, until it reaches a critical size, where the heat transfer is at maximum value. Beyond this critical size of the adiabatic object, will decrease the heat transfer rate. Das and Reddy [9] and Aminossadati and Ghasemi [10] studied conjugate natural convection in enclosures with a given inclination angle. They obtained the higher solid conductivity results in a better heat transfer and increasing the inclination angle improves the heat transfer performance.

Fu et al. [11] placed the isothermal cylinder object in the left half enclosure. They found a heat transfer enhancement by inserting a rotation object. Costa and Raimundo [12] considered a conductive rotating cylinder inserted in the center of a square enclosure. They concluded that the thermophysical properties of the cylinder object were important on the overall heat transfer process across the enclosure. Hussain and Hussein [13] analyzed effect of inserting a conductive rotating cylinder at different vertical locations inside a differentially heated square cavity. Besides, inserting the quadrilateral or cylinder objects, Shih et al. [14] inserted the adiabatic triangle object in the enclosure. They concluded that a bigger triangle size exhibits the highest heat transfer performance.

Present work aims to investigate the fluid flow and heat transfer characteristics for various polygon types and thermal properties placed inside the center of the square enclosure. Complete two-dimensional numerical simulation and systematical generalization of the conjugate heat transfer behavior occurring in the enclosure by varying the obstacles' shape is carried out.

## 2. Mathematical Formulation

**Figure 1:**
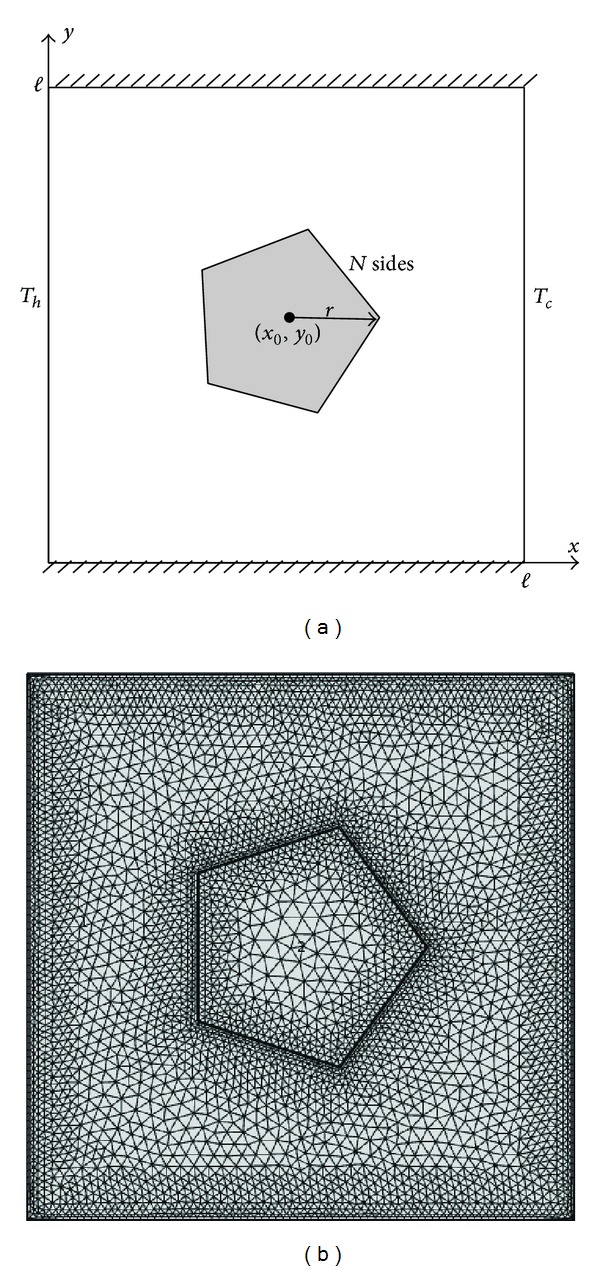
(a) Schematic representation of the model. (b) Mesh distribution.

Figure 1(a) presents the coordinate systems and a square enclosure having a conductive regular polygon placed at (*x*_0_, *y*_0_). It is assumed that the dimension in the *z*-direction is large enough and the end effects on the flow are negligible; that is, fluid flow and heat transfer are two-dimensional. The vertical walls of both enclosures are maintained constant and at uniform different levels of temperature, *T*_*h*_ and *T*_*c*_. The horizontal walls are kept insulated. Free space between polygons and walls enclosure are filled with the Newtonian fluid which is incompressible but expands or contracts with temperature changes. This assumption leads to the use of the Boussinesq approximation. Under the above assumptions, thermal radiation is neglected; the governing equations for steady natural convection flow using conservation of mass, momentum, and energy can be written as

(1) $$\begin{array}{c} \frac{\partial u}{\partial x}+\frac{\partial v}{\partial y}=0,\\ u\frac{\partial u}{\partial x}+v\frac{\partial u}{\partial y}=-\frac{1}{\rho }\frac{\partial p}{\partial x}+\nu \left(\frac{\partial^{2}u}{\partial x^{2}}+\frac{\partial^{2}u}{\partial y^{2}}\right),\\ u\frac{\partial v}{\partial x}+v\frac{\partial v}{\partial y}=-\frac{1}{\rho }\frac{\partial p}{\partial y}+\nu \left(\frac{\partial^{2}v}{\partial x^{2}}+\frac{\partial^{2}v}{\partial y^{2}}\right)+g\beta \left(T_{f}-T_{c}\right),\\ u\frac{\partial T_{f}}{\partial x}+v\frac{\partial T_{f}}{\partial y}=\alpha \left(\frac{\partial^{2}T_{f}}{\partial x^{2}}+\frac{\partial^{2}T_{f}}{\partial y^{2}}\right) \end{array}$$

and the energy equation for the solid polygon is

(2) $$\begin{array}{c} \frac{\partial^{2}T_{s}}{\partial x^{2}}+\frac{\partial^{2}T_{s}}{\partial y^{2}}=0, \end{array}$$

where the subscripts *f* and *s* stand for the fluid and the solid, respectively. No-slip condition is assumed at all the solid-fluid interfaces. Using the following nondimensional variables:

(3) X=xl,  Y=yl,  R=rl,  U=ulα,  V=vlα,Θf=Tf−TcTh−Tc,  P=pl2ρα2,  Pr=να,Ra=gβ(Th−Tc)l3Prν2,  Θs=Ts−TcTh−Tc.

The resulting nondimensional forms of (1)-(2) are

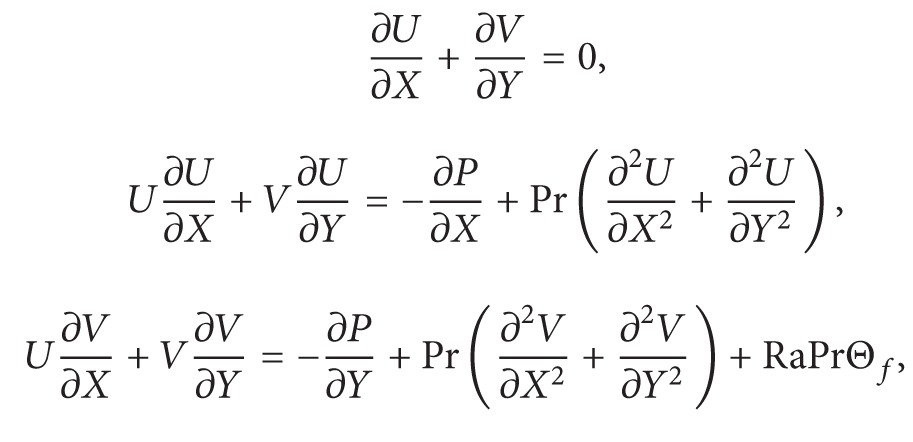
(4)

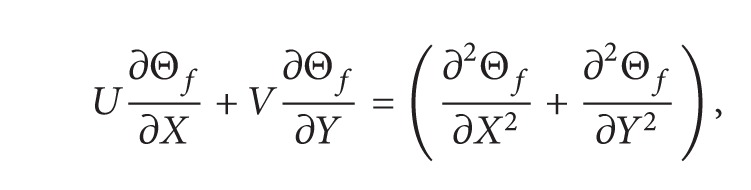
(5)

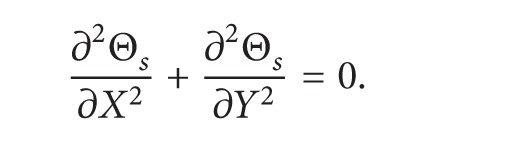
(6)
The values of the nondimensional velocity are zero in the solid region and on the solid-fluid interfaces. The boundary conditions for the nondimensional temperatures are:

(7) $$\begin{array}{c} \Theta_{f}=1\;\text{at}\;\;X=0,\\ \Theta_{f}=0\;\text{at}\;\;X=1,\\ \frac{\partial \Theta_{f}}{\partial Y}=0\;\text{at}\;Y=0,\;\;Y=1,\\ \Theta_{f}=\Theta_{s}\;\text{at}\;\text{the}\;\text{outer}\;\text{polygon}\;\text{surface},\\ \frac{\partial \Theta_{f}}{\partial \eta }=K_{r}\frac{\partial \Theta_{s}}{\partial \eta }\;\text{at}\;\text{the}\;\text{inner}\;\text{polygon}\;\text{surface}, \end{array}$$

where *K*_*r*_ = *k*_*s*_/*k*_*f*_ is the thermal conductivity ratio. All sides of the solid polygon are equal in length (regular polygon). Number of sides is denoted by *N*, where for *N* = 3 the shape is triangle, for *N* = 4 the shape is square, for *N* = 5 the shape is pentagon, for *N* = 6 the shape is hexagon, for *N* = 7 the shape is heptagon, for *N* = 8 the shape is octagon, and for *N* = *∞* the shape becomes cylinder. The polygon to enclosure area ratio is defined by

(8) $$\begin{array}{c} A=R^{2}N\frac{\sin\left(360/N\right)}{2}. \end{array}$$

The fluid motion is displayed using the stream function Ψ obtained from velocity components *U* and *V*. The relationships between the stream function and the velocity components are *U* = ∂Ψ/∂*Y* and *V* = −∂Ψ/∂*X*, which yield a single equation as

(9) $$\begin{array}{c} \frac{\partial^{2}\Psi }{\partial X^{2}}+\frac{\partial^{2}\Psi }{\partial Y^{2}}=\frac{\partial U}{\partial Y}-\frac{\partial V}{\partial X}. \end{array}$$

The physical quantity of interest in this problem is the heat transfer rate. The heat transfer rate distribution is obtained by applying Fourier's law at the hot wall; that is,

(10) $$\begin{array}{c} q=-{k_{f}\frac{\partial T_{f}}{\partial x}|}_{x=0} \end{array}$$

which becomes in terms of the dimensionless variables

(11) $$\begin{array}{c} \text{Nu}=\frac{ql}{\left(T_{h}-T_{c}\right)}=-{k_{f}\frac{\partial \Theta_{f}}{\partial X}|}_{X=0}, \end{array}$$

where Nu is local Nusselt number based on *l*. The average Nusselt number on the heated left wall is then found by integrating the local distributions; that is, by using

(12) $$\begin{array}{c} \bar{\text{Nu}}=\overset{1}{\underset{0}{\int }}-\frac{\partial \Theta_{f}}{\partial X}\text{d}Y. \end{array}$$

## 3. Computational Methodology

The governing equations along with the boundary conditions are solved numerically by the CFD software package COMSOL Multiphysics. COMSOL Multiphysics (formerly FEMLAB) is a finite element analysis, solver, and simulation software package for various physics and engineering applications. We consider the following application modes in COMSOL Multiphysics. The Incompressible Navier-Stokes application mode, (ns), the Convection-Conduction application mode (cc) and the Diffusion application mode (di) are used for equations (4), (5), and (6), respectively. P2-P1 Lagrange elements and the Galerkin least-square method are used to assure stability. A parallel direct solver (PARDISO) and the damped Newton method are implemented to solve the discretized equations. Here the convergence criterion is set to 10^−6^.

In this study, mesh generation on square enclosure containing polygon object is made by using triangles. The triangular mesh distribution is shown in Figure 1(b). Several grid sensitivity tests were conducted to determine whether the mesh scheme is sufficient and to ensure that the results are grid independent. We use the COMSOL default settings for predefined mesh sizes, that is, extremely coarse, extra coarse, coarser, coarse, normal, fine, finer, extra fine, and extremely fine. In the tests, we consider the parameters *N* = 5,  *X*_0_ = 0.5, *A* = *π*/25, Ra = 10^5^, and *K*_*r*_ = 1 as shown in Table 1. Considering the accuracy and CPU time, a finer mesh size was selected for all the computations done in this paper.

**Figure 2:**
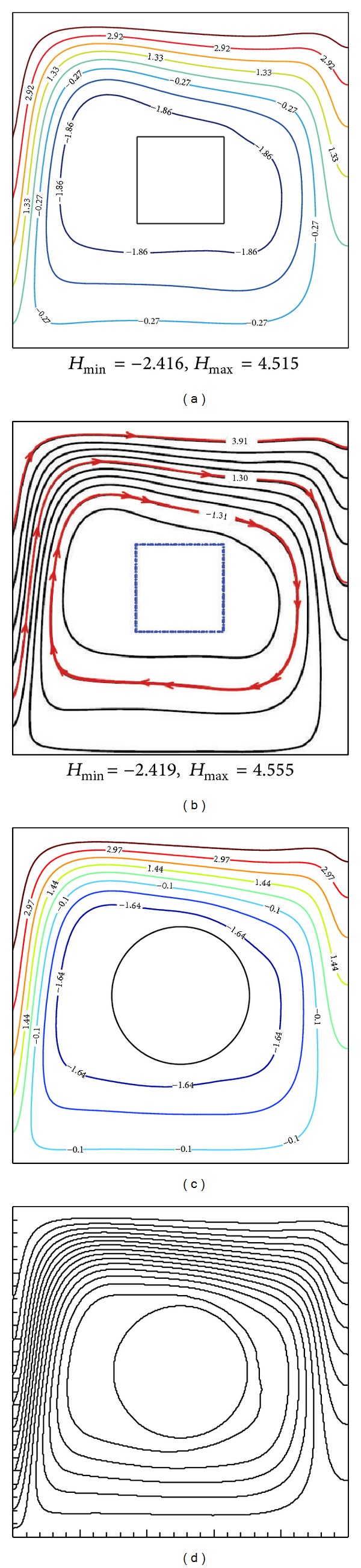
Comparison of the present computed heatlines (a, c) against that of Zhao et al. [5] for quadrilateral, *N* = 4 (b) and Costa and Raimundo [12] for cylinder, *N* = *∞* (d) at Ra = 10^5^, *X*_0_ = 0.5, and *K*_*r*_ = 1.

To validate the computational code, the previously published problems on conjugate natural convection in a square enclosure with a conducting quadrilateral and cylinder objects were solved.  Figure 2 shows the comparison of the present computed heatlines (a, c) against that of Zhao et al. [5] for the quadrilateral, *N* = 4 (b) and Costa and Raimundo [12] for the cylinder, *N* = *∞* (d) at Ra = 10^5^, *X*_0_ = 0.5, and *K*_*r*_ = 1. The comparison reveals good agreement with the reported studies. These comprehensive verification efforts demonstrated the robustness and accuracy of the present computation.

## 4. Results and Discussion

The analyses in the undergoing numerical investigation are performed in the following range of the associated dimensionless groups: the solid polygon to enclosure area ratio, 0 ≤ *A* ≤ *π*/16, the horizontal position, 0.25 ≤ *X*_0_ ≤ 0.75, the thermal conductivity ratio, 0.1 ≤ *K*_*r*_ ≤ 10, and the Rayleigh number, 10^3^ ≤ Ra ≤ 10^6^. The polygon shapes vary from triangle to cylinder, where cylinder is categorized as a special polygon having infinity sides.

**Figure 3:**
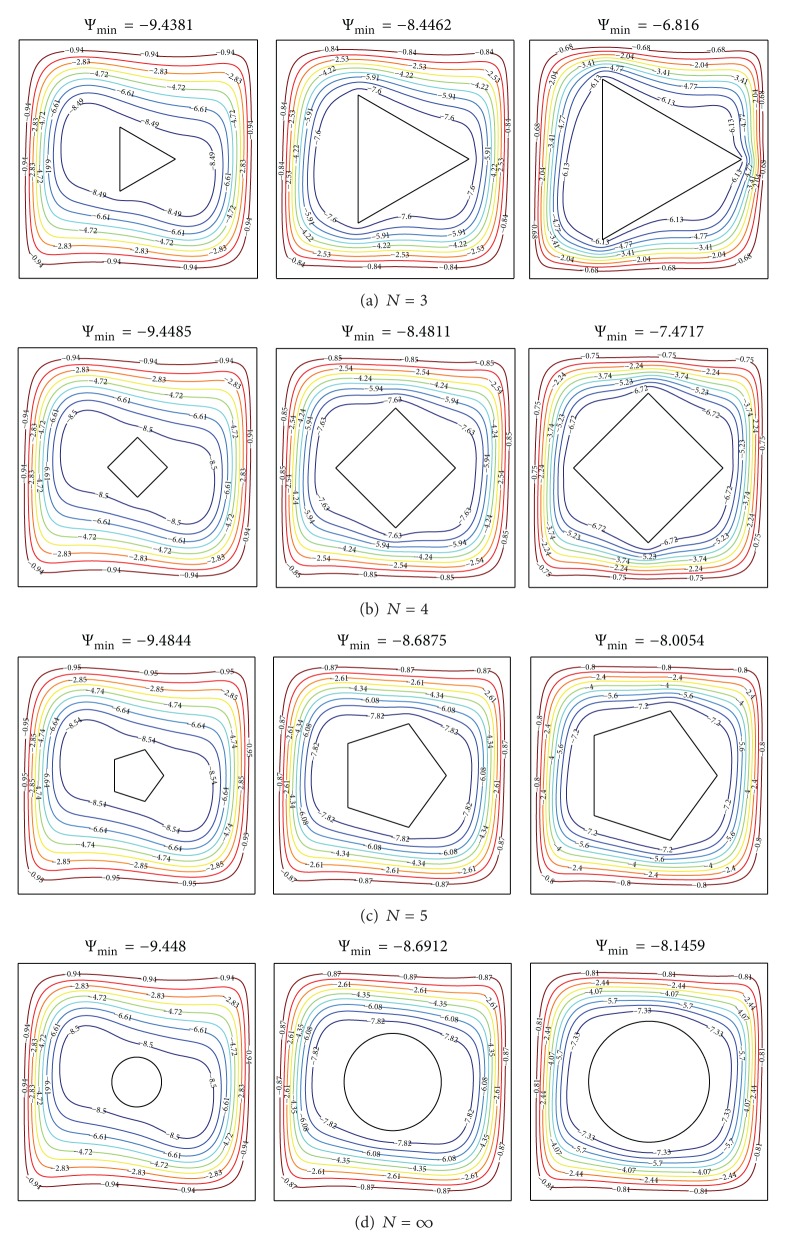
Streamlines for different polygon size *A* and number of polygon sides *N* at *K*_*r*_ = 1.0, *X*_0_ = 0.5, and Ra = 10^5^.

Figure 3 illustrates the streamlines for various types of solid polygon, where the solid area (*A*) attains the value *π*/100, *π*/25, and *π*/16, respectively. The thermal conductivity ratio is fixed at *K*_*r*_ = 1.0 and *X*_0_ = 0.5 and the Rayleigh number at Ra = 10^5^. The fluid temperature adjoining the hot surface rises and moves from the left to the right, falling along the cold surface, then rising again at the hot surface. This movement creates a clockwise circulation cell in free space between the polygon and walls enclosure. The cell shape adjacent to the outer polygon surface was bent by the presence triangle, quadrilateral and pentagon. The bent takes higher position as the polygon size is made bigger. The strength of the flow circulations decreases by increasing the solid area for the same polygon type. It is obvious that increasing the *A* leads to smaller space for the flow to circulate. At fixed *A*, increasing *N* increases the flow strength, as indicated from |Ψ|_min⁡_ values. This increasing occurs except at small cylinder, *A* = *π*/100, where the flow strength is weaker than the small solid pentagon inserted inside in the enclosure.

**Figure 4:**
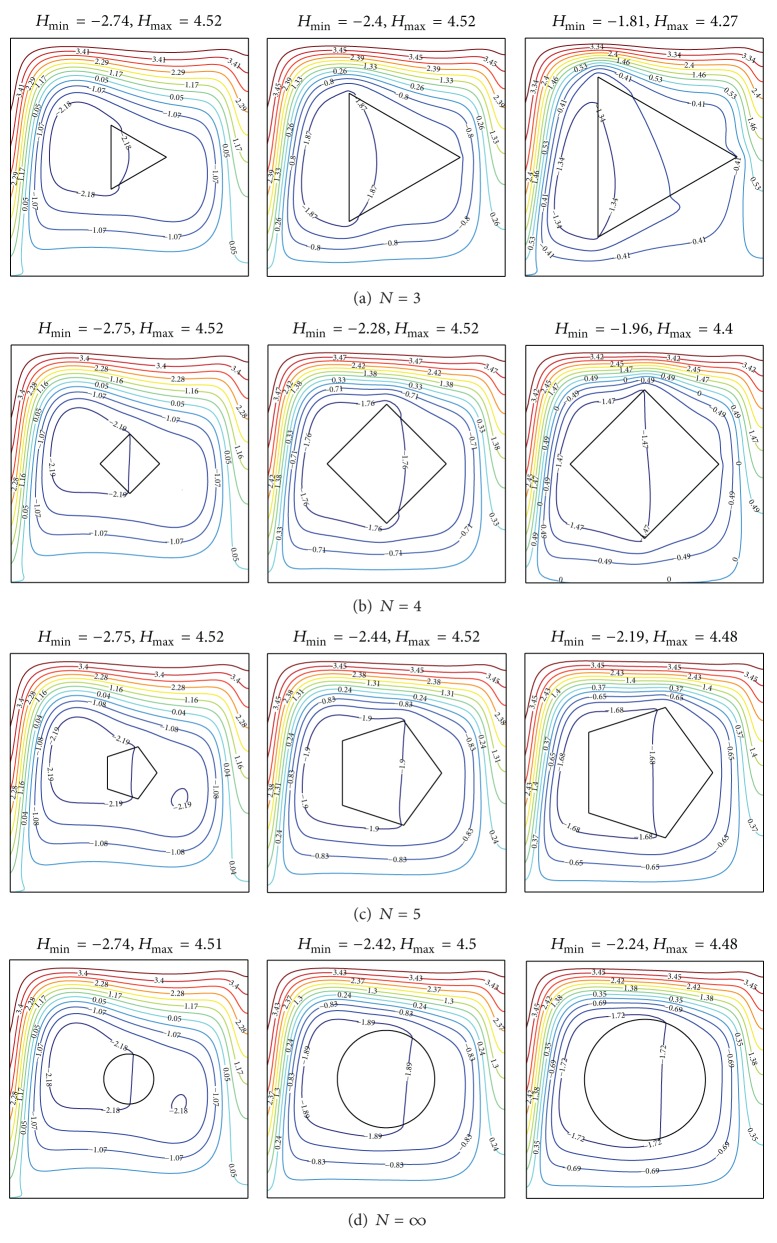
Heatlines for different polygons to enclosure area ratio *A* and number of polygon sides *N* at *K*_*r*_ = 1.0, *X*_0_ = 0.5, and Ra = 10^5^.

The corresponding heatlines, presented in Figure 4, show that open lines starting from the left hot surface and reaching the right cold surface are responsible for the heat exchange between the system and the environment. The heatlines crossing the hot surface are more crowded near the bottom side than those near the topside. Many of the heatlines follow the fluid flow. The heatlines indicate that heat is conducted vertically through the solid polygon. The closed-loop occurs at the various sizes and polygon types. We observed dual closed loop occurs in enclosure containing a small pentagon or a cylinder. The negative value in the closed-loop cells correspond to a passive zone in which heat rotates with small contribution to the heat transfer between the active walls. The bigger polygon sizes are observed to create greater suppression of the inner heat circulation as indicated from |*H*|_min⁡_ values. |*H*|_min⁡_ remains constant by increasing *N* at relative small solid area. Increasing *N* from 3 to 4 leads to a decreasing of the |*H*|_min⁡_ at medium size solid polygon. At relative large solid area, the |*H*|_min⁡_ values increases by increasing *N*. The *H*_max⁡_ values remain constant by adjusting the *N* and *A*. The *H*_max⁡_ is located at the upper part of the enclosure; this refers to the total amount of the system heat transfer.

**Figure 5:**
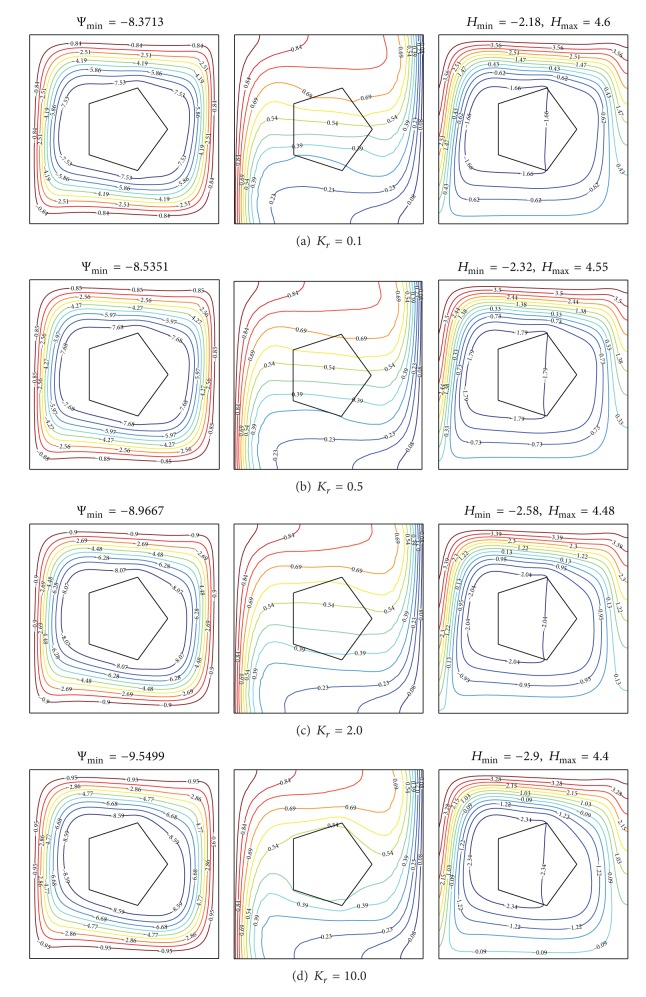
Streamlines (left), isotherms (middle), and heatlines (right) evolutions by varying thermal conductivity ratio for *N* = 5, *X*_0_ = 0.5, *A* = *π*/25, and Ra = 10^5^.

Figure 5 presents the streamlines, isotherms, and heatlines to show the effects of thermal conductivity ratio on flow fields, thermal and heat path characteristics for solid pentagon, *A* = *π*/25, *X*_0_ = 0.5, and Ra = 10^5^. The streamlines shown in the left of Figure 5 are all circulated as vortices, since there is no mass or fluid exchange between the system of the enclosure and its environment. The flow strength measured by |Ψ|_min⁡_ increases with increasing thermal conductivity ratio *K*_*r*_. The isotherms in the solid pentagon are sparser, while the isotherms in the fluid remain unchanged by increasing the solid conductivity. The inner heat circulation is enhanced when the solid conductivity is strengthened. The *H*_max⁡_ decreases as the solid conductivity is strengthened.

**Figure 6:**
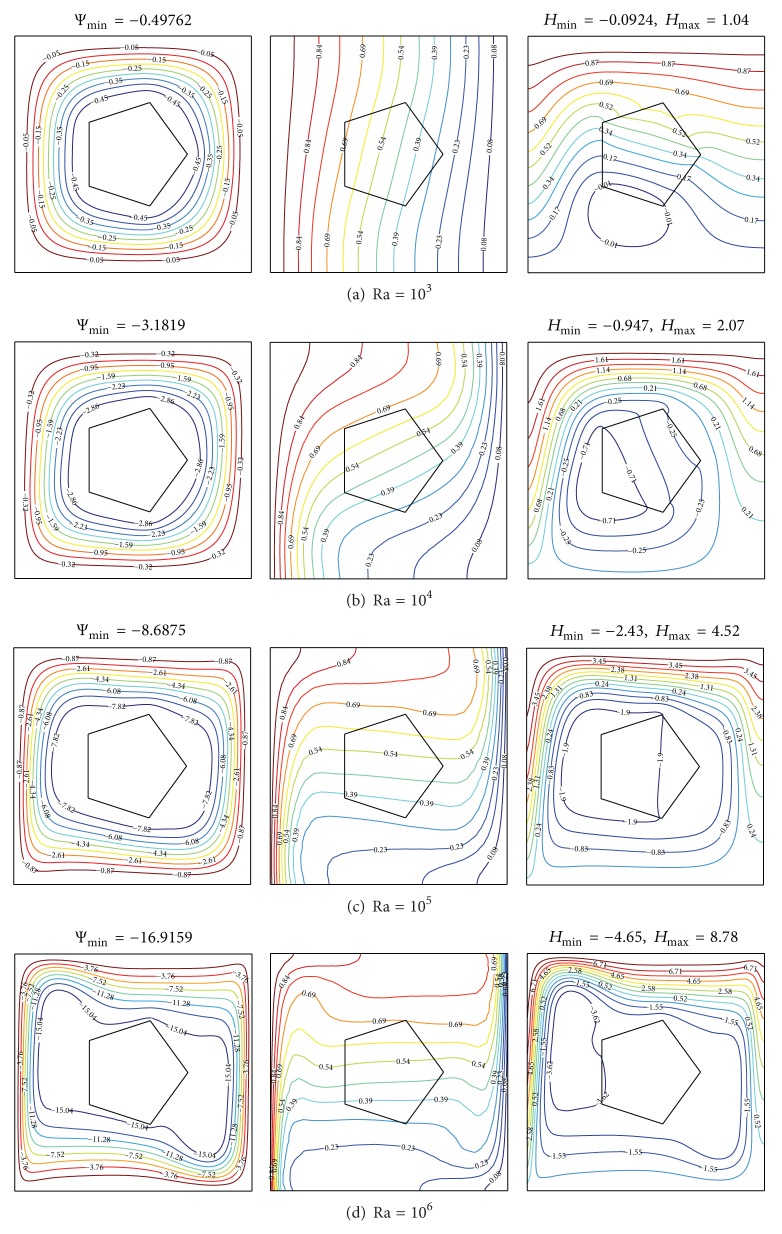
Streamlines (left), isotherms (middle), and heatlines (right) evolutions by varying Rayleigh number for *N* = 5, *X*_0_ = 0.5, *A* = *π*/25, and *K*_*r*_ = 1.0.

Figure 6 presents the streamlines, isotherms, and heatlines to show the effects of Rayleigh number on flow fields, thermal and heat path characteristics for solid pentagon, *A* = *π*/25, *X*_0_ = 0.5, and *K*_*r*_ = 1.0. At low Rayleigh number, the streamlines present a circular shape and are distributed uniformly. This is a signal of very weak convection. Consequently, the heat transfer across the enclosure is dominantly due to conduction process and because of that the heatlines and the isotherms both present pseudoconduction pattern. The flow strength measured by |Ψ|_min⁡_ increases with increasing the Rayleigh number. The temperature stratification is step by step developed. At Ra = 10^5^, the nonuniform distribution of streamlines is observed. The circular cells are elongated and indicate that convection is strong in the outer region and weak in the core. The isotherms in the solid pentagon are distributed horizontally, while the isotherms in the fluid at the isothermal walls are denser than before. The sparse heatlines in the solid pentagon indicate that the role of conduction is markedly weakened in the process of heat transfer. At strong buoyancy force, Ra = 10^6^, the streamlines are grouping in the thin outer region and sparse in the inner region. Crowded vertical thermal boundary layers are formed at isothermal walls. The flow circulation, inner heat circulation, and maximum heatlines values as presented in Figure 6 are enhanced as the buoyancy force is taken higher. The vortex of clockwise closed-loop cells moves from lower-middle region to upper-left region by increasing Ra.

**Figure 7:**
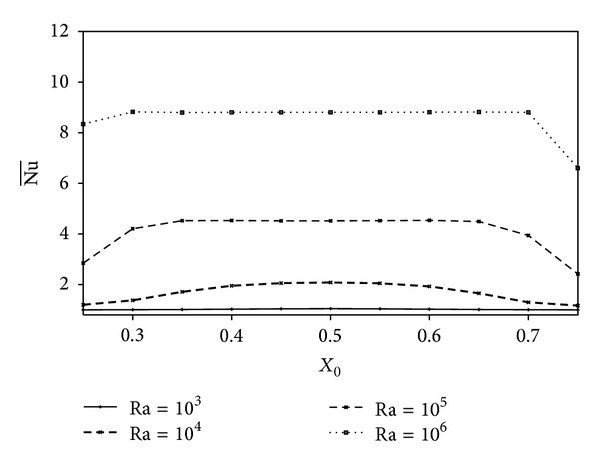
Variation of $\bar{\text{Nu}}$ with *X*_0_ for different values of Ra at *K*_*r*_ = 1.0 and *A* = *π*/25.

Variations of the average Nusselt number with the horizontal position *X*_0_ are shown in Figure 7 for *K*_*r*_ = 1.0, a polygon size, *A* = *π*/25, and different values of the Rayleigh number. The Nusselt number is seen to increase with increasing the Rayleigh number due to the increasing effect of convection. In general, when the inner polygon is displaced to the center of the cavity, the $\bar{\text{Nu}}$ increases due to the weakening of the interaction between the active walls and the solid polygon which becomes located outside the thermal boundary layers. For Ra = 10^3^, the heat transfer is dominated by conduction and the Nusselt number $\bar{\text{Nu}}$ remains unchanged by changing the polygon's position. For Ra = 10^4^, $\bar{\text{Nu}}$ presents a parabolic profile with a maximum value at *X*_0_ = 0.5. At Ra = 10^5^, $\bar{\text{Nu}}$ exhibits a flat profile in the range 0.35 ≤ *X*_0_ ≤ 0.65. This flat profile becomes wider for Ra = 10^6^, as a consequence of the development of the thermal boundary regime for this high value of Ra.

**Figure 8:**
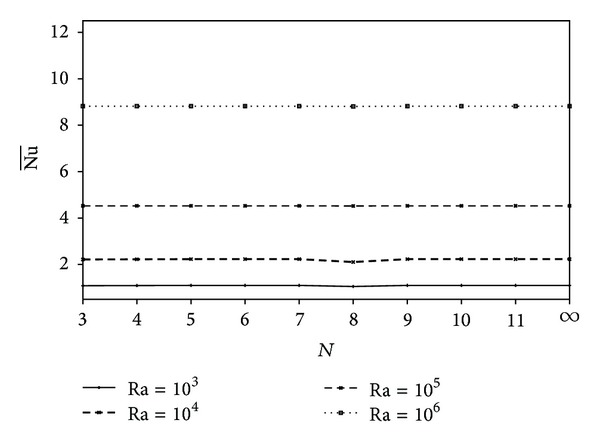
Variation of $\bar{\text{Nu}}$ with *N* for different values of Ra at *K*_*r*_ = 1.0, *X*_0_ = 0.5, and *A* = *π*/25.

Variations of the average Nusselt number with the number of polygon sides are shown in Figure 8 for different values of the Rayleigh number at *K*_*r*_ = 1.0 and *X*_0_ = 0.5 and polygon size, *A* = *π*/25. We applied the inverse of formula (8) to maintain the area of each polygon equivalent. Applying formula (8) to an input *R* and *N* gives a result of *A*; then applying the inverse formula (8) to *A* gives the result *R* and *N*. *R* and *N* are inversely proportional, a longer *R* will be used for a lower *N* or vice versa. Figure 8 shows that the average Nusselt number is unchanged by varying the polygon types at the considered values of Ra. Careful investigation reveals that the *H*_max⁡_ values shown in Figure 6 are equivalent with the $\bar{\text{Nu}}$ values in this figure. This is a proof that the overall heat transfer performance can be read directly from the positive heatline value at the upper part of the enclosure.

**Figure 9:**
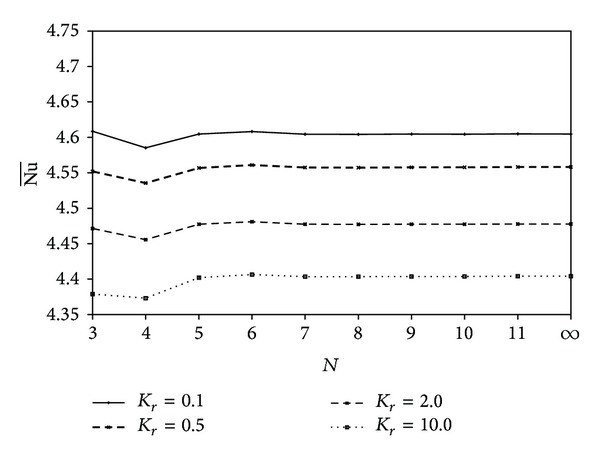
Variation of $\bar{\text{Nu}}$ with *N* for different values of *K*_*r*_ at Ra = 10^5^, *X*_0_ = 0.5, and *A* = *π*/25.

Variations of the average Nusselt number with the number of polygon sides are shown in Figure 9 for different thermal conductivity ratios at Ra = 10^5^, *X*_0_ = 0.5, and *A* = *π*/25. Increasing *N* from 3 to 4 leads to a decreasing of the average Nusselt number. This means that the square object leads to greater suppression of the inner heat circulation than the triangle object as indicated from the |*H*|_min⁡_ (see Figure 4). The average Nusselt number was shown to be stable for *N* ≥ 5. Increasing thermal conductivity ratio leads to a decrease of the Nusselt number for a fixed value of *N*.

**Figure 10:**
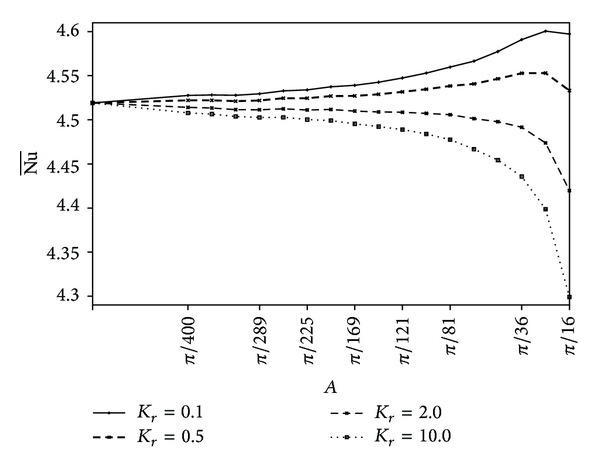
Variation of $\bar{\text{Nu}}$ with *A* for different values of *K*_*r*_ at Ra = 10^5^, *X*_0_ = 0.5, and *N* = 5.

Variations of the average Nusselt number with the polygon sizes are shown in Figure 10 for different thermal conductivity ratio at Ra = 10^5^, *X*_0_ = 0.5, and *N* = 5. Figure 10 exhibits the existence of a critical size of the solid pentagon at low conductivities, *K*_*r*_ = 0.1, 0.5; below which, the increase of the size increases the $\bar{\text{Nu}}$ and above which the increase of the size decreases the $\bar{\text{Nu}}$. The $\bar{\text{Nu}}$ is observed to decrease by increasing the solid size having high conductivities, *K*_*r*_ ≥ 2.0.

## 5. Conclusions

The present numerical simulations study the effects of various solid polygons properties on natural convection inside a square enclosure. The dimensionless forms of the governing equations were solved using the COMSOL Multiphysics software. Detailed computational results for fluid flow, temperature distribution, heat path, and heat transfer characteristics in the enclosure have been presented in graphical forms. The main conclusions of the present analysis are as follows.

1. The strength of the flow and inner heat circulations is much higher for greater *N*. The maximum heatlines value is not sensitive by changing the *N*.
2. The heat transfer rate remains stable for *N* ≥ 5 and polygons located at the center of the enclosure will assure the maximum heat transfer rate.
3. The critical size of the solid polygon was found exists at low conductivities. The heat transfer rate increases with the increase of the size of the solid polygon, until it reaches a maximum value. At this moment, the size of the solid polygon is reaches its critical value. Further, beyond this critical size of the solid polygon, will decrease the heat transfer rate.

The theoretical prediction in this paper is hoped to be a useful guide for the experimentalists to study the various combinations of the polygon shaped and its thermal conductivity properties to control the fluid flow and thermal performance of enclosure at different sizes. The factors of polygon location, orientation, and rotation with different angular speed will be the focus of our research undertaking.

## Figures and Tables

**Table 1:** Grid sensitivity check at *N* = 5, *X*_0_ = 0.5, *A* = π/25, Ra = 10^5^, and *K*_*r*_ = 1.

Predefined mesh size	Mesh elements	$\bar{\text{Nu}}$	CPU time (s)
Extremely coarse	261	4.4311	2
Extra coarse	459	4.4860	2
Coarser	677	4.5051	2
Coarse	1309	4.5101	2
Normal	1973	4.5120	2
Fine	2969	4.5162	2
Finer	8871	4.5193	3
Extra fine	24827	4.5195	6
Extremely fine	32411	4.5201	7

## Nomenclature

*A*: Polygon to enclosure area ratio
*g*: Gravitational acceleration
*K*_*r*_: Thermal conductivity ratio
*k*: Thermal conductivity
*l*: Width and height of enclosure
*N*: Number of polygon sides
$\overset{-}{\text{Nu}}$: Average Nusselt number
*r*&*R*: Radius of polygon & dimensionless radius of polygon
Ra: Rayleigh number
*T*: Temperature
*u*, *v*: Velocity components in the *x*- and *y*-directions
*x*, *y*&*X*, *Y*: Space coordinates & dimensionless space coordinates.

### Greek Symbols

*α*: Thermal diffusivity
*β*: Thermal expansion coefficient
Θ: Dimensionless temperature
*ν*: Kinematic viscosity.

### Subscript

*c*: Cold
*f*: Fluid
*h*: Hot
*s*: Solid.
